# Myocarditis, Coagulopathy, and Small Fibre, Sensory, and Multiple Cranial Nerve Neuropathy Complicating BNT162b2 Vaccination: A Case Report

**DOI:** 10.7759/cureus.55205

**Published:** 2024-02-29

**Authors:** Josef Finsterer

**Affiliations:** 1 Neurology, Neurology and Neurophysiology Center, Vienna, AUT

**Keywords:** multiple cranial nerve neuropathy, myopericarditis, small fibre neuropathy, adverse reaction, sars-cov-2 vaccination

## Abstract

SARS-CoV-2 vaccinations can lead to complications, including post-acute COVID-19 vaccination syndrome (PACVS). There has been no report of a patient with PACVS presenting with Guillain-Barre syndrome (GBS), myocarditis/pericarditis, immunodeficiency, or coagulopathy after the second BNT162b2 dose.

The patient is a 51-year-old woman with chronic myopericarditis, coagulopathy due to factor-VIII increase and protein-S deficiency, GBS, and a number of other ocular, dermatological, immunological, and central nervous system abnormalities related to the second dose of the BNT172b2 vaccine. GBS manifested with mild, multiple cranial nerve lesions, small fibre neuropathy (SFN) affecting the autonomic system with postural tachycardia syndrome (POTS) and orthostatic hypotension, and sensory disturbances in the upper and lower limbs. PACVS was diagnosed months after onset, but despite the delayed diagnosis, the patient benefited from glucocorticoids, repeated HELP apheresis, and multiple symptomatic treatments.

The case shows that SARS-CoV-2 vaccination can be complicated by PACVS manifesting as chronic myopericarditis, coagulopathy, GBS with predominant dysautonomia, and impaired immune competence, and that diagnosis of PACVS can be delayed for months. Delayed diagnosis of PACVS may result in a delay in appropriate treatment and the prolongation of disabling symptoms. Patients and physicians should be made aware of PACVS to improve diagnostic and therapeutic management in terms of patient and healthcare system costs.

## Introduction

Multiple non-industry-sponsored studies, case series, and case reports have shown that any of the commercial anti-SARS-CoV-2 vaccines can be harmful and even fatal to individual patients of any ethnicity [1]. Adverse reactions following SARS-CoV-2 vaccination can occur within hours or a few days after vaccination (acute COVID-19 vaccination syndrome (ACVS)) or several days or months after vaccination (post-acute COVID-19 vaccination syndrome (PACVS)) [2]. The side effects of SARS-CoV-2 vaccinations particularly affect the central and peripheral nervous systems (CNS, PNS) [3], the cardiovascular system [1], the endocrine system, the gastrointestinal tract, the kidneys [4], the immune system, or the coagulation system [5]. One of the PNS complications of SARS-CoV-2 vaccinations is Guillain-Barre syndrome (GBS) [6]. GBS can have different subtypes, such as acute, inflammatory demyelinating polyneuropathy (AIDP), acute motor (and sensory), axonal neuropathy (AM(S)AN), Miller Fisher syndrome (MFS), mono- or polyneuritis of cranial nerves (CNs), pharyngeal-cervico-brachial (PCB) weakness, brainstem Bickerstaff encephalitis (BBE), and dysautonomia due to affection of small nerve fibres (small fibre neuropathy [SFN]) [7]. All of these subtypes can overlap. The most common causes of GBS besides SARS-CoV-2 are *Campylobacter jejuni*, *Hemophilus influenza*, *Mycoplasma pneumoniae*, herpes, Epstein-Barr, cytomegaly, varicella zoster, hepatitis E, respiratory syncytial, or Zika viruses. Clinical presentation, diagnostic management, and therapeutic management of SARS-CoV-2 vaccine-associated GBS do not differ from GBS due to other causes.

The most common cardiac complications of SARS-CoV-2 vaccinations are myocarditis and pericarditis. mRNA-based, vector-based, and DNA-based vaccines can cause myocarditis [8]. Myocarditis and pericarditis can be complicated by heart failure, systolic dysfunction, and supraventricular or ventricular arrhythmias. There are also some reports of valvar and non-valvar endocarditis following SARS-CoV-2 vaccinations [9]. The two most common coagulopathies caused by SARS-CoV-2 vaccinations are vaccine-induced thrombocytopenia and thrombosis (VITT) and disseminated intravascular coagulopathy (DIC) [5]. There has been no report of a patient with PACVS manifested by GBS with cranial and autonomic nerve impairment, myocarditis/pericarditis, and coagulopathy after vaccination with an mRNA-based anti-SARS-CoV-2 vaccine.

## Case presentation

The patient is a 51-year-old Caucasian woman, height 167 cm, weight 50 kg, with a history of symphysial arthrosis and uncomplicated migraine, who was diagnosed with PACVS 2.5 years after onset. The family history was positive for dilated cardiomyopathy (father). PACVS began to develop one month after the second vaccination with the BNT162b2 vaccine. She received the first dose of BNT162b2 in April 2021, followed by a day of fatigue. After receiving the second dose of BNT162b2 in June 2021, she developed fatigue for one day and a rash on the chest and abdomen for two days. Three weeks after the second vaccination, she suffered rhinoconjunctivitis, followed by rhinosinusitis. Since July 2021, she has developed undulating fatigue that has persisted to date (Table 1). At the same time, she also developed undulating orthostasis, which has persisted to this day. In September 2021, there was further deterioration with new symptoms such as exertional dyspnea, shortness of breath, dry cough, chest pain, palpitations, tachycardia, arterial hypotension, excessive blood pressure variability, dysarthria, hoarseness, muscle tension, exercise intolerance, nausea, and loss of appetite, leading to a weight loss of 8 kg (Table 1). This was accompanied by difficulty concentrating, brain fog, memory problems, loss of multitasking ability, sleep disorders, sensitivity to noise and light, and a feeling of Globus (“chroot”) (Table 1). Since November 2021, she has been having carpopedal spasms at night.

**Table 1:** Dynamics of symptoms developing or resolving over a period of 2.5 years since the second dose of BNT162b2 until the end of January 2024. PEM: post-exertional malaise, CNS: central nervous system, PNS: peripheral nervous system, RR: blood pressure. *Cell values refer to the month/year in which a symptom was present or absent.

Symptom	Present	Absent
General		
Fatigue	7/21−1/24*	Never
Exercise intolerance	9/21−1/24	Never
PEM	9/21−8/22, 12/22−1/24	9/22−11/22
Nausea	9/21−11/22, 1/23, 4−5/23, 7/23, 9−11/23	12/22, 2−3/23, 6/23, 8/23, 12/23−1/24
Loss of appetite	9/21−11/22, 3−4/23, 9/23−1/24	12/22−2/23, 5−8/23
Sore lymph nodes	1/22−1/24	6/21−12/21
Rhinoconjunctivitis	6−7/21, 9−10/21, 06/22, 08−11/22, 10/23	Rest of time
Cardiovascular		
Exertional dyspnea	9/21−1/24	Never
Pressure feeling on chest	9/21−11/22, 1−2/23, 5/23, 8−11/23, 1/24	12/22, 3−4/23, 6−7/23, 12/23
Shortness of breath	9/21−9/22, 1−3/23, 8−10/23, 12/23−1/24	10−12/22, 4−7/23, 11/23
Dry cough	9−12/21, 1/23	1−12/22, 2/23−1/24
Anginal chest pain	9/21−11/22, 1−2/23, 4/23, 6−7/23, 10−11/23, 1/24	12/22, 3/23, 5/23, 8−9/23, 12/23
Palpitations, tachycardia	9−12/21, 3−12/22, 6/23	1−2/22, 1−5/23, 7/23−1/24
Arterial hypotension	9−12/21, 5−10/22, 6/23, 11−12/23	1−4/22, 11/22−5/23, 7−10/23, 1/24
Extensive RR variability	9−12/21, 4/22, 9/22, 11/22, 10/23	1−3/22, 5−8/22, 10/22, 12/22−9/23, rest
PNS		
Orthostasis	7/21−1/23, 3−4/23, 6/23−1/24	2/23, 5−23
POTS	3/22, 7/22, 10/22−1/23, 4/23, 6/23, 8/23	Most likely never
Presyncope	10/22, 6/23, 10/23	Rest of time
Dysarthria	9/21−1/24	Never
Hoarseness	9/21−1/24	Never
Paresthesias of tongue, mouth	6−10/22, 12/22, 2−3/23, 5/23−1/24	6/21−5/22, 11/22, 1/23, 4/23
Facial palsy (right)	12/22−2/23, 5/23, 8/23	6/21−11/22, 3/23−4/23, 6/23−7/23, rest
Numbness right face	2/23, 9−10/23, 1/24	6/21−1/23, 3−8/23, 11−12/23
Chewing weakness	3−4/22, 6/22, 12/22	6/21−2/22, 5/22, 7−11/22, 1/23−1/24
Paresthesias of neck	12/21−1/22, 6/22, 11/22, 2/23, 5−7/23, 12/23−1/24	Rest of time
Hypertension of axial muscles	9/21−9/22, 11/23−1/24	10/22−10/23
Hypertense respiratory muscles	9−12/21, 3/22, 6−7/22, 10/22, 12/22−2/23, 5/23, 7/23, 9/23, 11−12/23, 01/24	Rest of time
Fasciculations (legs, lips, tongue)	4−8/22, 7/23−1/24	6/21−3/22, 9/22−1/24
Weakness of hands	6−7/22, 11/22−2/23, 6/23, 12/23−1/24	6/21−5/22, 8−10/22, 3−5/23, 7−11/23
Carpopedal spasms	11/21−5/23	6−10/21, 6/23−1/24
Hypoesthesia of hands	2/23, 5/23−1/24	6/21−1/23, 3−4/23
Hypoesthesia of feet	6−8/22, 12/22, 2/23, 5/23−1/24	6/21−5/22, 9−11/22, 1/23, 3−4/23
Paresthesias of feet	6−10/22, 2/23	Rest of time
Heat intolerance	6/22−1/24	6/21−5/22
CNS		
Headache	3/22−9/22, 11/22−12/23	6/21−2/22, 10/22, 1/24
Impaired concentration	9/21−1/24	Never
Brain fog	9/21−1/24	Never
Memory impairment	9/21−1/24	Never
Loss of multitasking ability	9/21−1/24	Never
Oversensitivity to light and noise	9/21−1/24	Never
Sleep disorder	9/21−1/24	Never
Globe feeling, throat tightness	9/21−1/24	Never
Eyes		
Blurred vision	4−8/22	6/21−7/22, 1/23−1/24
Dermatologic		
Exanthema	6/21, 12/21, 12/22	Rest of time
Blue toes	12/21, 12/22−2/23	Rest of time

**Figure 1:**
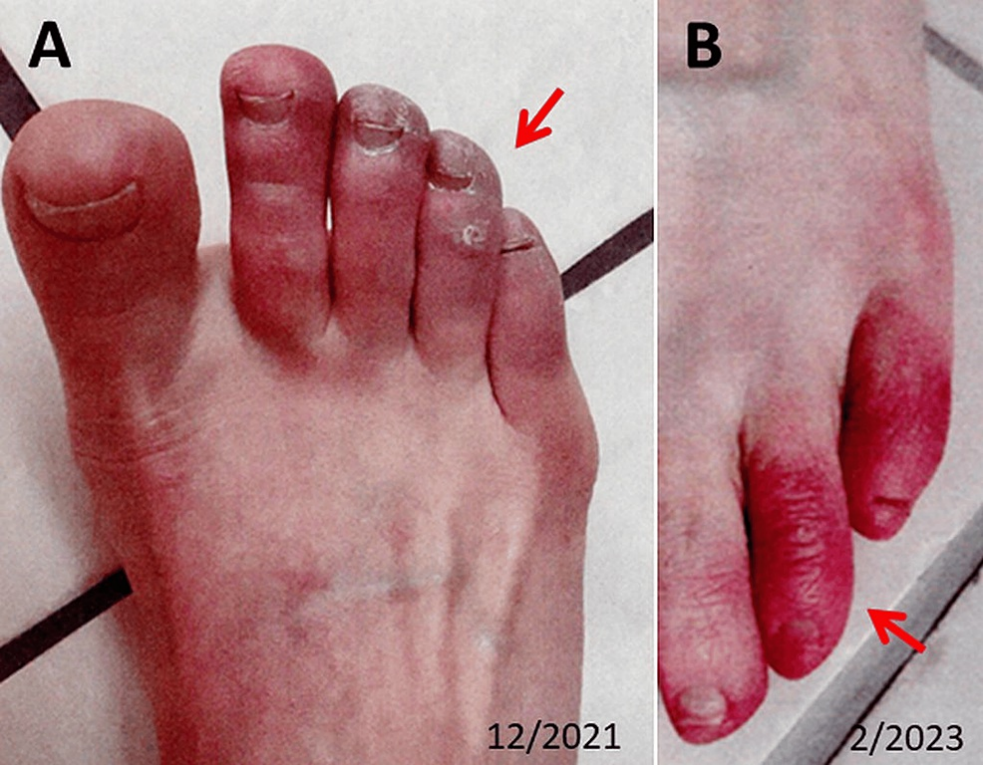
COVID toes (arrow) were first identified in December 2021 (panel A). Recurrence of COVID toes in February 2023 (arrow) after the COVID-19 infection (panel B).

**Figure 2:**
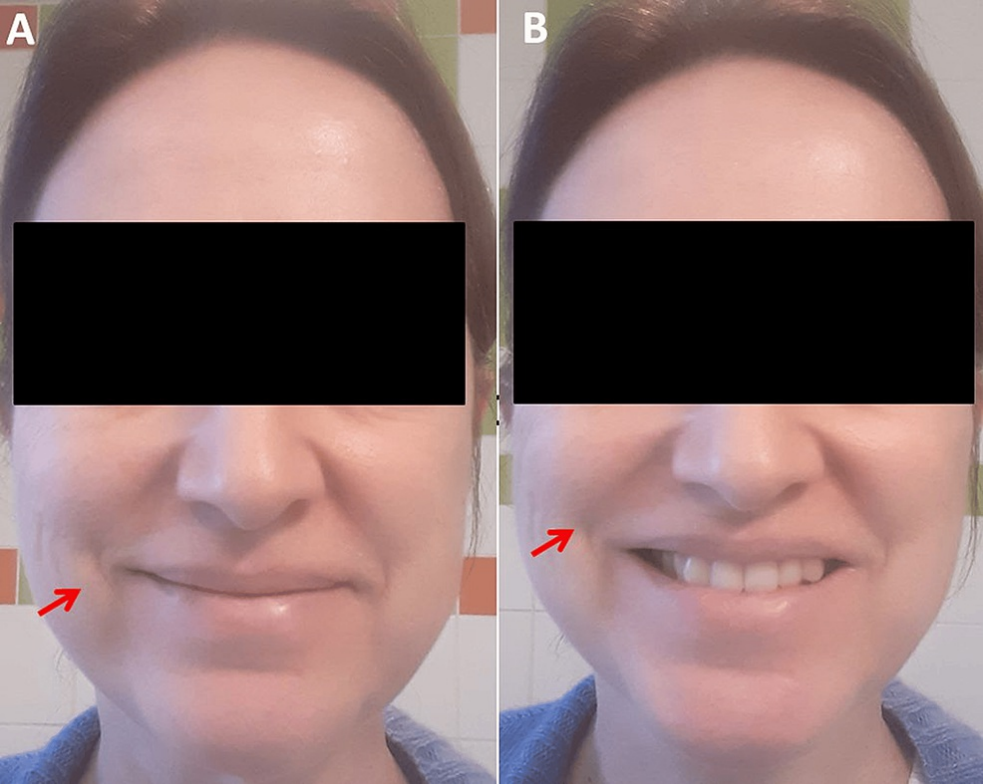
The index patient’s face shows minimal hypo-innervation of the right inferior branch of the facial nerve (arrow, panel A, and panel B) and mild ptosis on the left side (panel B), both of which were absent before vaccination.

After receiving the third dose of BNT1621b2 in December 2021, she experienced worsening fatigue, chest pain, and sleep disturbances, as well as a transient rash of the chest and abdomen, chilblains, and blue toes (Figure 1). In March 2022, a new-onset herpes labialis occurred with headaches and paresthesias in the head area. In June 2022, she also developed hypoesthesia, paresthesia, pain in both feet, fasciculations of lower extremity muscles, and hypoesthesia in both hands. In December 2022, she first noted mild, right-sided temporary facial palsy (Figure 2). Table 1 summarises the progression of these symptoms up to January 2024. Despite her three vaccinations, she suffered a mild SARS-CoV-2 infection in December 2022, as documented by a positive RT-PCR.

**Figure 3:**
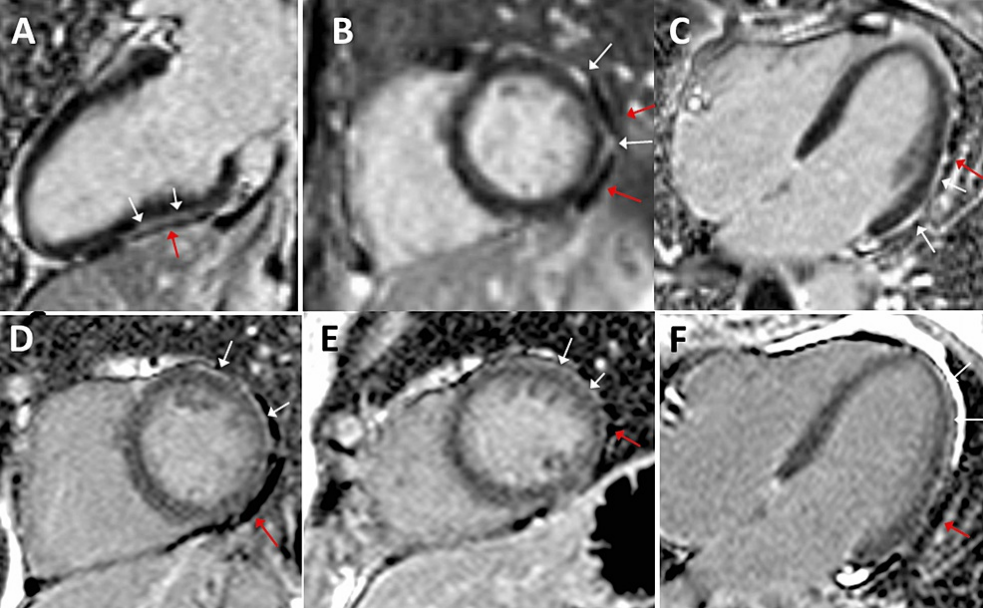
Cardiac MRI in June 2022 (panels A-C) showing late gadolinium enhancement in the basal, apical, anterolateral, and inferolateral segments (white arrows) and pericardial effusion (red arrows). These abnormalities were still present on a follow-up MRI in October 2023 (panels D-F).

An initial cardiologic examination using ECG, long-term ECG, and echocardiography in October 2021 only revealed mild ascending ST depression, mild mitral valve prolapse, and mild mitral, aortic, and tricuspid insufficiencies. Myocardial scintigraphy revealed exertional ischemia of the apex, anterior and posterior walls, and septum. Coronary angiography and right heart catheter examination for questionable pulmonary hypertension were normal. Cardiac MRI showed mild enlargement of the right atrium and ventricle, as well as epicardial late gadolinium enhancement (LGE) in the left ventricular myocardium and mild pericardial effusion (Figure 3). The follow-up echocardiogram in December 2022 was unchanged from the previous one. A follow-up cardiac MRI in October 2023 still revealed LGE in the same distribution as before, mild diastolic dysfunction, microvascular angina, and aortic root fibrosis (Figure 3). proBNP and troponin were normal. Chronic myopericarditis was diagnosed. Pulmonary examination diagnosed exertional dyspnea, mild hyperventilation, discrete air trapping, mild elevation of biomarkers of bronchial inflammation, and chronic fatigue syndrome (CFS). Spiroergometry was normal.

**Figure 4:**
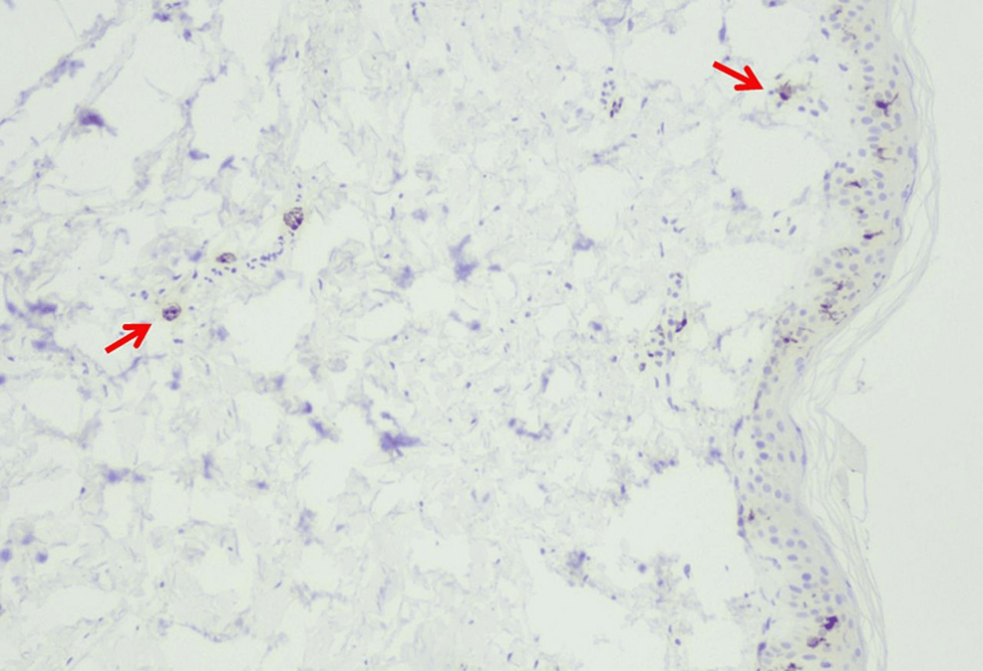
Skin biopsy from the left ankle shows reduced number of intraepidermal nerve fibres (arrows) of 6/mm using the modified HRP-polymer method (panel B). ^The modified HRP-polymer method has been described by Bakkers et al. [10].

Repeat neurological examinations were either normal or revealed impaired concentration, dysarthria, dysphonia, fasciculations, decreased tendon reflexes in the lower limbs, hypoesthesia of the hands and feet, paresthesia of the feet, pallhypaesthesia of the lower limbs, and autonomic neuropathy. Autonomic neuropathy manifests as postural tachycardia syndrome (POTS), pre-syncope, decreased baro-receptor sensitivity, sudomotor dysfunction with hypohidrosis, delayed bladder emptying, insomnia, and orthostatic intolerance. She collapsed during a Schallong test. A cerebral MRI showed mild, nonspecific microangiopathy. The carotid ultrasound was normal. Nerve conduction studies, repetitive nerve stimulation, needle electromyography, and visually evoked potentials were normal. F-wave responses were absent on both peroneal nerves. Median somato-sensory-evoked potentials (SSEPs) were normal but showed prolonged latencies of cortical responses after tibial nerve stimulation. The sudomotor axon reflex was abnormal bilaterally. Quantitative sensory testing revealed dysfunction of the A-delta and C-fibres of the upper and lower limbs but a normal function of the A-beta and A-gamma fibres. Cerebrospinal fluid (CSF) examinations revealed that CSF protein was in the upper normal range (423 mg/l [n = 150-450 mg/l]). Oligoclonal bands were negative. Antineuronal antibodies were repeatedly normal. Immuneencephalitis-associated antibodies were negative. A skin biopsy on the left distal lower limb showed significantly reduced fibre density (Figure 4). Therefore, SFN was diagnosed. Oto-rhino-laryngological examinations revealed protracted rhinosinusitis and hypofunctional dysphonia. An initial fiberoptic endoscopic examination of swallowing (FEES) revealed incomplete glottic closure. Stroboscopy revealed glottal insufficiency, hypotrophic vocal folds, and reduced vocal fold elasticity. A second FEES showed deterioration of glottal closure insufficiency during phonation, decreased left vocal fold mobility, and ary cartilage dysdiadochokinesia. The examiner also described mild right facial palsy and fasciculations of the lips and tongue. Orthopaedic examination revealed genua valga, generalised hypermotility, mild scoliosis, adductor irritation, retropatellar pain, and atlas blockade.

Coagulopathy evaluation in January 2023 revealed transiently elevated factor VIII (174% [n, 70-150%]) and decreased protein S antigen (57% [n, 65-115%]) with normal activity. Thromboelastography in December 2023 still showed premature clotting, but two months later, factor VIII and protein S levels were back to normal. Hereditary coagulopathy was excluded. She had no history of coagulopathy before the SARS-CoV-2 vaccination. Serum iron was reduced. TSH was occasionally slightly elevated. IgE was repeatedly elevated. Intracellular ATP was reduced, and mitochondrial mass increased. Immunological workup revealed questionable long-EBV (reactivation) syndrome with borderline EBV IgM and elevated IgG early antigen, elevated CMV IgG and IgM, immunologic dysfunction of T-cells (low CD3-, CD56-, CD57-, and NK-cells), reduced TH1- and TH2-cell response, reduced TH1/TH2 ratio of 1.3 (n, 6.1-21), and reduced IFN-gamma production of TH1 cells. TNF-alpha, IL-4, IL-6, and IL-10 were also reduced. Antibodies against ganglionic acetylcholine receptor a3 were positive. Among the neuronal antibodies, she was slightly positive for SOX1 twice. Beta2-AAK (beta-autoantibodies) were also positive. CCP-AK-IgG was slightly increased. ANA was increased once to 1:320 and C3 was reduced, but rheumatological disease could be ruled out. Myositis antibody testing revealed borderline-positive YARS (Ha) antibodies. PACVS serum markers such as AT1R, ETAR, IL-1-Rb, alpha2b-adr-R, betal2-adr-R, M2R antibodies, and IL-8 were increased. In addition to CFS and SFN, multisystem inflammatory syndrome (MIS) was diagnosed.

After right heart catheterisation in December 2022, she received enoxaparin for 10 days. Prednisone had a short-term, beneficial effect on some of her symptoms in March 2022 and February 2023. She underwent 10 heparin-induced extracorporeal LDL precipitation (HELP) apheresis between October 2022 and March 2023 and started on ASS plus Clopidogrel due to suspicion of MIS. Despite these measures, a catheter thrombosis occurred during the second HELP apheresis in October 2022 and three months later in January 2023, which required evaluation for coagulopathy. After detection of increased factor VIII and decreased protein S, unfractionated heparin was added to ASS and clopidogrel. Since she no longer tolerated heparin well, it was replaced by apixaban in addition to clopidogrel in April 2023. Additionally, statin therapy was initiated. HELP apheresis had a subjective positive effect on fatigue, tinnitus, dysphonia, and hypoesthesia in the hands and feet. Orthopedists prescribed naltrexone for myalgia. She is currently taking prednisolone (5 mg/d), apixaban (5 mg/d), clopidogrel 75 mg/d, acetyl-salicylic acid 100 mg/d, ivabradin (10 mg/d), rosuvastatin (15 mg/week), losartan (37.5 mg/d), coenzyme-Q10 (200 mg/d), magnesium (300 mg/d), vitamin-D (0.025 mg/d), naltrexone (4.5 mg/d), valacyclovir (1500 mg/d), inosin/dimepranol-4-acetamidobenzoat (1500 mg/d), lysin (3200 mg/d), nattokinase (6000 FU/d), quercetin (500 mg/d), sulodexide (250 ULS/d), N-acetylcystein (1800 mg/d), ginko biloba (240 mg/d), melatonin (2 mg/d), and copper (3 mg/d).

## Discussion

Several reasons make the patient's presentation interesting. First, it shows that SARS-CoV-2 vaccinations can be complicated by multisystem disease, affecting the brain, peripheral nerves, heart, coagulation system, and immune system. Cerebral complications may include headaches, difficulty concentrating, memory impairment, brain fog, increased irritability, and difficulty sleeping. Peripheral nerve involvement may include SFN, cranial nerve lesions, sensory neuropathy, fasciculations, and muscle hypertension. Due to impairment of the immune system, new or recurring, previously unknown infections or allergies can occur. An impairment of the coagulation system can manifest itself in hypercoagulability. Cardiac side effects of vaccination may include myocarditis, pericarditis, and arrhythmias. General symptoms may include fatigue, exercise intolerance, post-exertional malaise, loss of appetite, nausea, and painful lymph nodes. Second, the case shows that PACVS symptoms can vary significantly over time. Third, establishing the diagnosis of PACVS can be delayed for months due to misinterpretation of symptoms and instrumental findings, a lack of binding diagnostic criteria for PACVS, and a delay in the application of appropriate treatment. A PACVS diagnosis can be supported by a PACVS antibody profile suggestive of PACVS [11].

Although all complications diagnosed in the index patient have been previously reported, the combination of cardiac, neurologic, coagulation, and immune system complications is unique. Pericarditis and SFN have been previously reported [12]. Hypercoagulation in the form of VITT, complicated by multiple thromboses or bleeding, has also been reported [13]. There are also cases of myocarditis plus coagulopathy after the SARS-CoV-2 vaccination [14]. Immunosuppression is also a common vaccination complication. Arguments for immunosuppression in the index patient were recurrent rhinosinusitis, herpes labialis, probable EBV reactivation, new allergies, and the deflected immunological parameters. All of these findings suggest limited immune competence after vaccination. The coagulopathy in the index patient was transient as factor VIII and protein S levels normalised on follow-up measurements. However, despite normal coagulation parameters, the patient was recommended to continue triple therapy to prevent the recurrence of the hypercoagulability described twice during apheresis. An increase in factor VIII and a decrease in protein S could be an indication of the formation of so-called fibrin amyloid microclots (fibrinaloids), which may be involved in the pathophysiology of PACVS [15]. These microclots can persist, entrap other proteins, and lead to the production of various autoantibodies [15]. Triple anticoagulation therapy can remove these microclots [15]. In keeping with this, the patient reported an improvement in her condition under triple anticoagulant therapy.

The diagnosis of GBS was based on the clinical presentation and instrumental findings. GBS manifested as multiple cranial nerve neuropathy, sensory neuropathy, and SFN in the index patient. Arguments for the involvement of multiple cranial nerves include recurrent right-sided facial palsy (CN VII), recurrent right facial hypoesthesia (CN V), dysarthria, dysphonia, and dysphagia (CN IX, X), fasciculations of the tongue (CN XII) and lips (CN VII), and paresthesias of the tongue (CN V). Cranial nerve involvement in PACVS most commonly affects the facial nerve [16], but all other nerves can also be affected [17]. Although cranial nerve involvement in PACVS was only mild and missed for months in the index patient, it was obvious and confirmed repeatedly by multiple physicians. Although a classical dissociation between CSF cell count and CSF protein could not be documented, GBS was very likely based on the clinical presentation and the borderline normal CSF protein. The absence of classic “dissociation cyto-albuminique” can be explained by the delayed CSF examinations. Another argument for GBS, in addition to cranial nerve involvement, was the multilocular fasciculations and the missing F-waves of the peroneal nerve. GBS manifesting as SFN has occasionally been reported [18]. There are also reports about autonomic involvement in SARS-CoV-2 vaccination-related GBS [19]. Further arguments for GBS in the index patient are the positive effects of steroids and HELP apheresis on general, cardiovascular, and neurological symptoms. The beneficial effect of apheresis in PACVS-associated GBS has been reported in a few cases [20]. Their beneficial effects have been attributed to improved microcirculation and the reduction of fibrinogen through apheresis. Intravenous immunoglobulins (IVIGs) have not yet been used because they have not been approved by the health insurance company.

## Conclusions

The presented case shows that SARS-CoV-2 vaccination can be complicated by chronic myocarditis, coagulopathy, impaired immune competence, GBS manifested by multiple cranial nerve lesions, and SFN (dysautonomia and sensory neuropathy). The case also shows that the diagnosis of PACVS can be delayed because the causal relationship between many different symptoms may not be immediately recognised, and some findings may be misinterpreted. A delay in the correct diagnosis may result in a delay in appropriate treatment and the prolongation of disabling symptoms. Patients and physicians should be made aware of PACVS to improve diagnostic and therapeutic management in terms of patient and healthcare system costs.
